# THE SIGNIFICANT ROLE OF HOUSING COORDINATION IN CONNECTICUT’S MONEY FOLLOWS THE PERSON PROGRAM

**DOI:** 10.1093/geroni/igz038.944

**Published:** 2019-11-08

**Authors:** Kathy G Kellett, Dawn Lambert, Tamara Lopez, Julie Robison

**Affiliations:** 1 UConn Health, Farmington, Connecticut, United States; 2 Connecticut Department of Social Services, Hartford, Connecticut, United States; 3 University of Connecticut, Farmington, Connecticut, United States

## Abstract

Housing coordination, a central component of the Money Follows the Person program, allows older adults and people with disabilities to experience greater independence and sense of well-being (Butler & Cabello, 2018; Koenig, 2015). This study was part of the 2017 Money Follows the Person (MFP) Process Evaluation involving 26 key informants (KIs) who completed telephone interviews sharing their experiences about program implementation. Of these, 13 KIs providing housing coordination services to the MFP transition team were asked about the housing coordination training they received and suggestions to improve the training. They were also asked about housing resources, how they develop their housing inventory, and recommendations for a “Housing Best Practices Report.” Interviews were audio-taped and transcribed. Data were analyzed using ATLAS.ti. Results demonstrate a need for more training and assistance to enable housing coordinators to expand their knowledge of housing alternatives, increase their awareness of housing policy/process changes, and further inform consumers about housing choices. Suggestions to improve housing coordination included offering more creative solutions during monthly housing coordination phone calls. Housing inventory challenges mentioned by KIs included time constraints, limited staff, administrative delays, and lack of affordable housing. Suggestions for housing inventory development focused on improving the management/maintenance of housing inventories. Best housing practices underscored the importance of communicating, teaming, building relationships with landlords and management companies, and standardizing housing policies and procedures. Overall recommendations included strengthening collaboration among housing coordinators to identify and implement best practices and improving housing inventory development to widen housing options for consumers.

